# Extracellular vesicles: an emerging tool for wild immunology

**DOI:** 10.1093/discim/kyae011

**Published:** 2024-06-24

**Authors:** Camila Espejo, Vanessa O Ezenwa

**Affiliations:** Department of Ecology and Evolutionary Biology, Yale University, New Haven, CT, USA; Department of Ecology and Evolutionary Biology, Yale University, New Haven, CT, USA

**Keywords:** wild immunology, comparative immunology, extracellular vesicles

## Abstract

The immune system is crucial for defending organisms against pathogens and maintaining health. Traditionally, research in immunology has relied on laboratory animals to understand how the immune system works. However, there is increasing recognition that wild animals, due to their greater genetic diversity, lifespan, and environmental exposures, have much to contribute to basic and translational immunology. Unfortunately, logistical challenges associated with collecting and storing samples from wildlife, and the lack of commercially available species-specific reagents have hindered the advancement of immunological research on wild species. Extracellular vesicles (EVs) are cell-derived nanoparticles present in all body fluids and tissues of organisms spanning from bacteria to mammals. Human and lab animal studies indicate that EVs are involved in a range of immunological processes, and recent work shows that EVs may play similar roles in diverse wildlife species. Thus, EVs can expand the toolbox available for wild immunology research, helping to overcome some of the challenges associated with this work. In this paper, we explore the potential application of EVs to wild immunology. First, we review current understanding of EV biology across diverse organisms. Next, we discuss key insights into the immune system gained from research on EVs in human and laboratory animal models and highlight emerging evidence from wild species. Finally, we identify research themes in wild immunology that can immediately benefit from the study of EVs and describe practical considerations for using EVs in wildlife research.

## Graphical Abstract

**Graphical Abstract:**
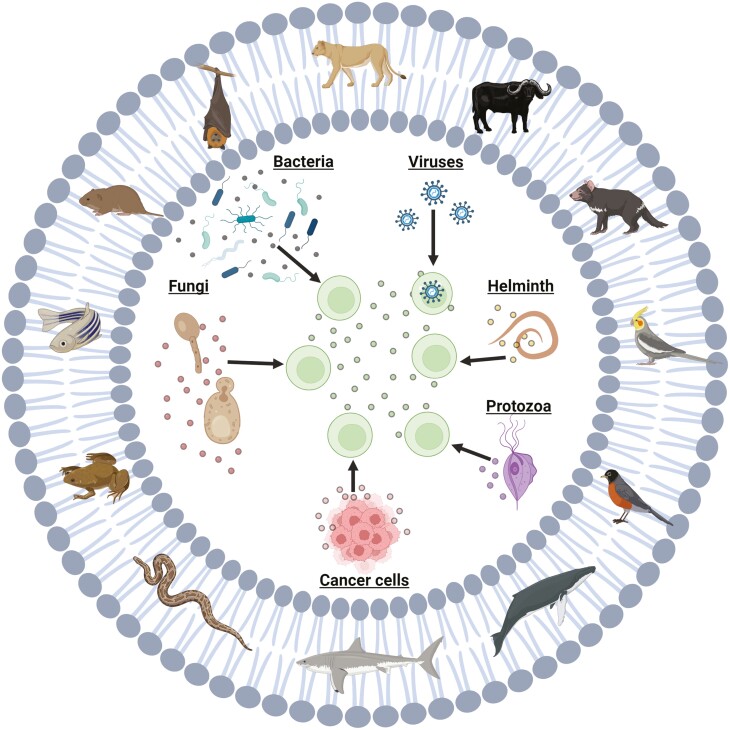

## Introduction

The vertebrate immune system is a complex network of cells and molecules that evolved, in part, to identify and neutralize pathogens. Given the near constant threat of pathogen infection all animals face, understanding how the immune system works and how it can be exploited to minimize pathogen-associated morbidity and mortality has been a driving force in immunology [1]. To achieve this goal, traditional research in immunology relies heavily on laboratory animal models to study immune system complexities [2]. These models have yielded fundamental insights into immunobiology, such as validating clonal selection theory in rats, the concept at the basis of modern immunology that when an antigen enters the body, it binds to a matching lymphocyte clone (B or T cells), triggering lymphocyte proliferation and the deployment of an army of identical cells to fight that specific antigen [3]. Despite such advances, it is increasingly recognized that lab animal models do not fully capture the diversity and complexity of immune system responses observed in humans and other animals [4, 5]. This ‘translation gap’ is attributable, at least in part, to the lack of variation that characterizes laboratory animals. The models of choice in traditional immunology are typically genetically homogeneous and naïve to real-world environmental challenges, both of which can constrain immune variation, limiting the translatability of laboratory model-based observations to humans and non-laboratory animals [6, 7].

Due to the limitations of traditional laboratory animal models and aided by new molecular and quantitative tools, the study of wild animal immune systems—termed ‘wild immunology’—has gained momentum over the past two decades [8–12]. This emerging approach studies immune responses of genetically diverse animals in their natural environments. In this subfield of immunology, variability beyond the laboratory is not perceived as ‘noise’, rather, it represents the ‘real world’, where continuous biotic (e.g. food competition, predator encounters, microbial exposures) and abiotic (e.g. seasonality) forces shape immune system function and evolution [5]. Supporting this view, a recent comparison of traditional lab mice to wild mice showed that immune profiles of lab mice resembled a human neonatal state, whereas wild mice immune profiles were more reminiscent of adult humans [13]. Such findings highlight the importance of incorporating organisms in natural environments into a broader view of immunobiology [5].

**Figure 1:**
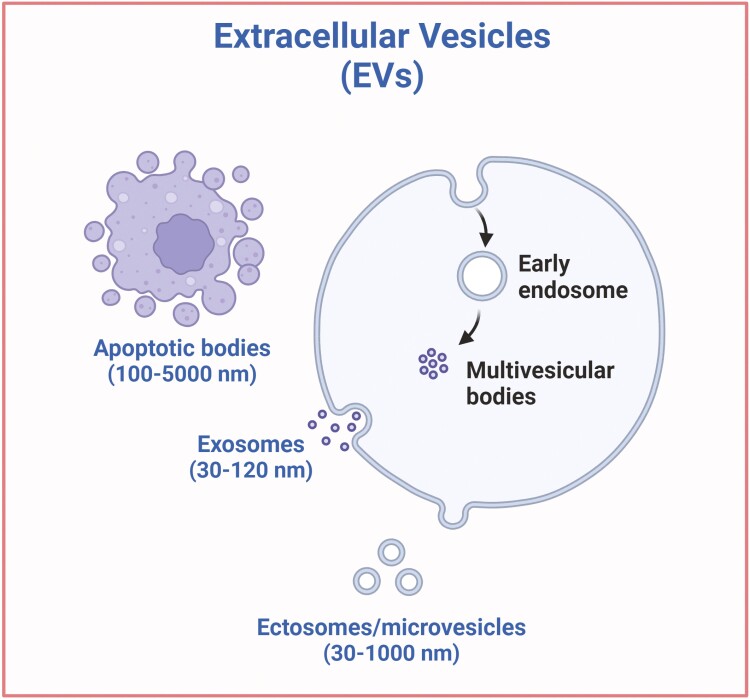
extracellular vesicle biology. Cells release three main types of extracellular vesicles (EVs): exosomes, microvesicles (also known as ectosomes), and apoptotic bodies. Exosomes are formed through the fusion of multivesicular bodies with the plasma membrane. Ectosomes emerge from plasma membrane budding, while apoptotic bodies are produced from cellular fragmentation during apoptosis [15]. Different EV subtypes overlap in size and lack distinct transmembrane and cytosolic protein markers; therefore, current guidelines recommend using the general term ‘EV’ [14]

Despite the promise of wild immunology, studying the immune systems of non-model species comes with many challenges, including the lack of commercially available, species-specific reagents and methods [the ‘reagent gap’ [11]. While ‘omics’ techniques (e.g. transcriptomics, proteomics) have begun to bridge this gap, progress remains slow because many non-model species lack sequenced genomes, hindering full utilization of these methods [11]. Logistical constraints related to sample collection and storage in field settings can further impede collection of relevant immune data in wild animal populations. One potential avenue to circumvent many of these challenges is the study of extracellular vesicles (EVs). EVs are membrane-bound, non-replicating nanoparticles, released by virtually all cell types [14]. EVs are broadly classified into three subtypes based on how they are formed (exosomes, ectosomes or microvesicles, and apoptotic bodies [herein referred to collectively as ‘EVs’ [14, 15]; Fig. 1]), and a growing body of research highlights the pivotal role of EVs in intercellular communication [16]. Within the immune system, EVs are significant because they contribute to orchestrating immune responses. This includes roles in activating and regulating immune cells and as essential messengers in immune signaling [17]. EVs have also been isolated from a range of vertebrate body fluids, including blood, urine, saliva, milk and feces [18], allowing accessible sample collection from wild species. In this review, we explore the untapped potential of EVs for studying immunological processes in wild animals. First, we review current understanding of EV biology across diverse organisms. Then, we describe key insights about the immune system derived from research on EVs in human and laboratory animal models. Next, we identify research themes and questions in wild immunology that can immediately benefit from the study of EVs. Lastly, we consider key advantages and disadvantages of employing EVs in wildlife research.

## Extracellular vesicles are universal and conserved across the tree of life

**Figure 2:**
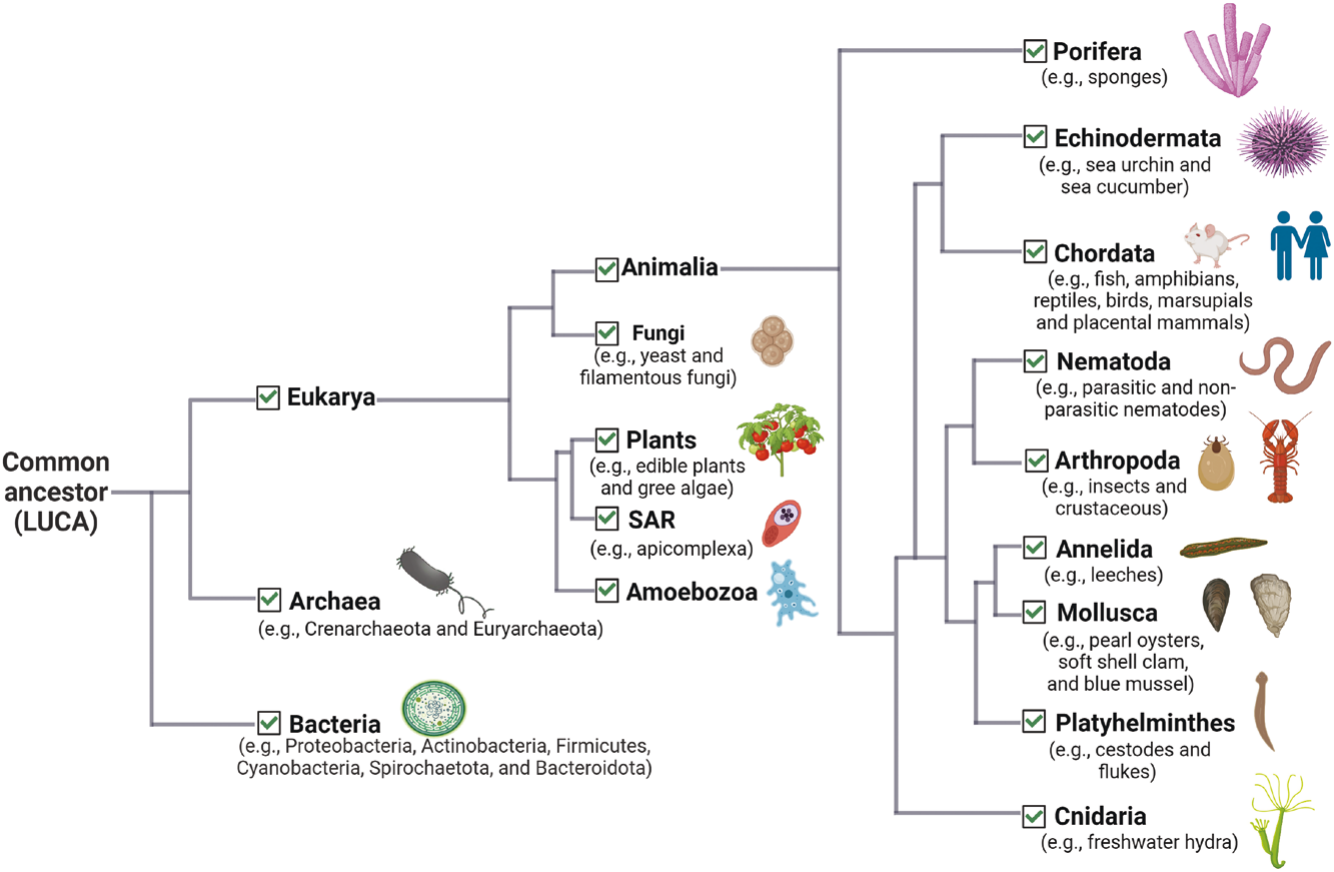
distribution of extracellular vesicles across the tree of life. EVs have been documented across all three biological domains, including a wide diversity of eukaryotes. Tree topology is based on the TimeTree of Life [TToLL5 [117]. Branch lengths are for illustrative purposes only and are not to scale

EVs contribute to intercellular communication by transporting bioactive molecules such as proteins, lipids, and nucleic acids between cells at various distances [16]. Research across all biological domains confirms that EVs are produced by organisms in every domain (Fig. 2), suggesting a conserved cell-to-cell communication mechanism [19]. The universality of EV secretion suggests a common origin, potentially tracing back to ancient lipid vesicles from the ‘primordial soup’ preceding the earliest known cellular forms [20]. In the harsh primordial environment, primitive lipid vesicles likely underwent structural refinements for enhanced robustness and better protection of their RNA contents [21]. By the era of the Last Universal Common Ancestor (LUCA), EV production mechanisms were likely already in play, setting the stage for the current forms of EV-mediated intercellular communication observed today [19].

Among eukaryotes, EV formation and release have been documented in protists, fungi, plants, and animals [19]. In animals, EVs have been detected in a variety of species, including fish [22], shellfish [23], insects [24], nematodes [25], reptiles [26], birds [27], marsupials [28], and placental mammals [29–31]. In some cases, the molecular contents of these particles are also similar across certain taxa. For instance, several small non-coding RNAs (microRNAs) critical for post-transcriptional regulation, are consistently packaged in EVs derived from the milk of diverse mammals [32]. Ultimately, the observed degree of consistency in EV presence across diverse organisms suggests that for non-model organisms, EVs may offer a promising avenue for studying core biological processes, including immune function.

## Immunological insights from extracellular vesicles

Research on laboratory animal models and humans has linked EVs to various immune phenomena, including lymphocyte maturation, fetal immune tolerance, allergic reactions, among others (see Table 1). Here, we focus on EV-related insights into innate and adaptive immunity and host-pathogen interactions, topics that are relevant for wild immunology. Innate immunity, acts as a first line of defense against pathogens, and involves non-specific responses such as barrier protection and phagocytosis [41]. By contrast, adaptive immunity develops more slowly in response to specific pathogen exposures, and offers highly specific pathogen targeting and memory [41]. How organisms deploy these immune responses varies depending on host (e.g. sex, age, genotype), pathogen type (e.g. virus, bacteria, worm), and other factors. Highlights from human and lab animal research offer clues as to how EVs can be used to advance understanding of such immune variation in wild species.

**Table 1:** examples of extracellular vesicle involvement in immune system communication

EV source (tissue/cell type)	EV target (cell/tissue type)	Impact of EVs on the target cell/tissue	Species	Key studies
Thymic epithelial cells 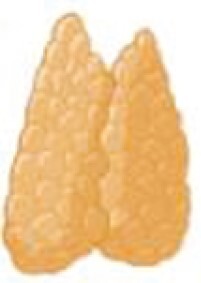	Immature T cells	Promote lymphocyte maturation and thymic egress	Mouse	[33]
Placental cells 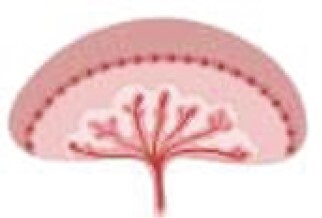	Natural killer and T cells	Confer immune tolerance to fetus	Human	[34, 35]
Dendritic cells 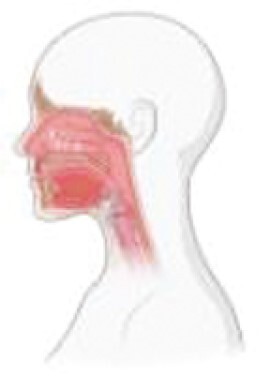	T cells	Present cat antigens and induce allergic response	Human	[36]
Mammary gland cells 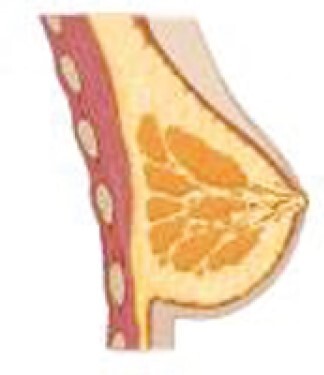	T cells	Suppress T cells without inducing tolerance	Human	[37]
Endothelial cells 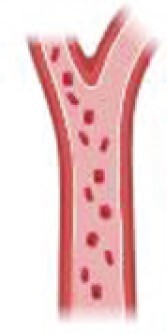	B cells	Stimulate autoantibody production and graft rejection	Mouse	[38, 39]
Astrocytes 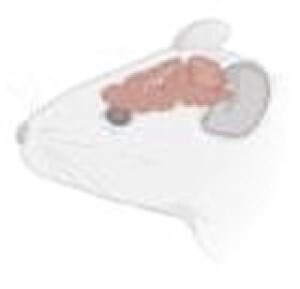	Liver cells	Stimulate peripheral immune cell migration to the brain post-injury	Mouse	[40]

### EVs in innate and adaptive immunity

Inflammation, crucial in the innate immune response, is a defense mechanism that is conserved across organisms [42]. Triggered by pathogens, damaged cells, and toxic compounds, inflammation removes harmful elements and initiates the healing process [41]. Research shows that EVs are instrumental in the inflammatory process [17]. For example, a mouse study found that EVs originating from neutrophils transfer arachidonic acid to platelets, and platelets use this acid to synthesize thromboxane A2, which promotes vasoconstriction and platelet aggregation, thereby facilitating neutrophil migration into inflamed tissues [43]. EVs also carry diverse immune mediators (e.g. microRNAs, cytokines, plasma proteins, complement proteins) that regulate innate responses and serve as indicators of the immunological profile [17]. For instance, in a study involving humans and rodents, EVs containing the complement receptor protein C5aR1 released by neutrophils were associated with reduced C5aR1 expression and altered neutrophil function [44]. Therefore, EV profiles can serve as potential markers of immune dysfunction. EVs also play a pivotal role in the functioning of B and T lymphocytes, the key mediators of the adaptive immune system. For example, EVs derived from B cells in humans and mice carry major histocompatibility complex (MHC) molecules that present antigens to T cells [45]. Similarly, antigen-presenting cells, especially dendritic cells, transfer MHC-antigen complexes to T cells via EVs [46]. Moreover, EVs were found to contain a higher concentration of MHC complexes and an over-representation of certain post-translationally modified MHC peptideligands compared to their originating cells [47]. These findings suggest that EVs could be a useful tool for exploring MHC variability in wild animals, providing much needed insight into MHC peptide-binding diversity and specificity.

### Host–pathogen interactions and EVs

Beyond contributing to a basic understanding of the immune system, research on EVs has also provided insight into how the host immune system recognizes pathogens and how pathogens, in turn, modulate host immunity. For example, bacteria produce membrane vesicles (called outer membrane vesicles [OMVs] in gram-negative bacteria and membrane vesicles [MVs] in gram-positive bacteria), that carry microbe-associated molecular patterns (MAMPs) [48]. Upon bacterial invasion, MAMPs are detected by pattern recognition receptors on host immune cells, triggering an immune response [49]. For instance, OMV-associated lipopolysaccharides from *Salmonella typhimurium* activate mouse dendritic cells via Toll-like receptor 4, leading to dendritic cell maturation and production of proinflammatory cytokines [50]. EVs associated with protozoan and helminth parasites also stimulate the host immune system. For example, although the malarial parasite *Plasmodium falciparum* does not directly produce EVs, in humans, it induces infected red blood cells (iRBCs) to release EVs that contain *Plasmodium* DNA [51]. When these iRBC-derived EVs enter monocytes, they activate the STING (Stimulator of Interferon Genes) pathway, leading to production of type 1 interferon and other cytokines [51]. Similarly, for *Brugia malayi*, the nematode responsible for lymphatic filariasis in humans, EVs released by third-stage larvae are internalized by murine macrophages *in vitro*, inducing a classically activated macrophage phenotype [52]. These studies suggest that EVs play a central role in orchestrating the host immune response to various pathogen challenges, a function that makes EVs a promising tool for identifying immune biomarkers of disease.

While pathogen derived EVs can stimulate the host immune system facilitating recognition and defense against pathogens, they are also exploited by pathogens to promote their own survival and replication within the host. One example is the gram-positive bacterium *Mycobacterium tuberculosis*, which secretes MVs that transport lipoglycans to host T cells, inhibiting CD4+ T-cell activation, thereby allowing the bacterium to escape host immune surveillance [53]. EVs from helminth parasites and cancer cells also carry immunoregulatory molecules that induce host immunosuppression. For instance, EVs from the cestode *Echinococcus granulosus*, when cultured with murine peripheral mononuclear cells, inhibit CD4+ and CD8+ T-cell proliferation in a dose-dependent manner [54], potentially aiding parasite establishment and development [54]. Similarly, EVs derived from tumor cells carry immunoregulatory molecules, such as Programmed Cell Death ligand1 (PDL1) and Fas lignd (FASL), that contribute to cancer by inhibiting T-cell function [55]. These examples highlight a key pathogen strategy: the use of EVs to evade the host immune system and suggest the significant potential of using EVs to understand how pathogens manipulate host immunity.

## EVs in wild immunology

Results from lab animal and human studies illustrate the promise of EVs for wild immunology. In particular, these studies suggest the key EV function of transporting immune-associated molecules can be exploited to study how wild immune systems respond to diverse challenges in natural settings. Indeed, emerging work on wildlife EVs is already revealing the utility of this approach. For example, recent studies in reindeer, seabirds, cetaceans, and sea lamprey have used EVs to identify deaminated proteins, a group of modified proteins that play important roles in various immunological and disease processes [27, 29, 56, 57]. Below, we explore four thematic areas where EVs hold promise for advancing wild immunology: (i) improving disease detection and diagnostics, (ii) understanding pathogenesis, (iii) characterizing mechanisms of host resistance and tolerance, and (iv) unraveling pathways by which co-infecting pathogens interact.

### Disease diagnostics

Early and accurate wildlife disease diagnosis is of growing importance, especially given the rise in disease emergence events [58]. Common diagnostic methods largely rely on direct detection of pathogen material (e.g. amplification of pathogen DNA via PCR) or indirect detection of pathogen exposure based on the host immune response (e.g. quantification of antibodies via serological techniques) [59]. However, these tests can lack accuracy due to a combination of factors, including low levels of pathogen DNA in host tissues and an inability to distinguish current infection from past exposure with antibody responses. In human medicine, EV-based diagnostic approaches show promise for diagnosing various diseases [60]. For instance, the glycolipid, lipoarabinomannan (LAM), a cell wall component of *Mycobacterium tuberculosis* bacteria, is an emerging candidate biomarker for tuberculosis (TB) diagnosis [61]. Because LAM is excreted in urine, urine-based LAM quantification holds promise as a rapid and sensitive TB testing tool. While a range of methods have been used to detect LAM in urine, a recent study using immuno-PCR detection of LAM in urinary EVs reported better sensitivity compared to studies using ELISA or lateral-flow immunochromatography for detecting LAM in neat urine [62]. Similarly, EVs may provide new opportunities for more sensitive wildlife disease detection.

**Figure 3:**
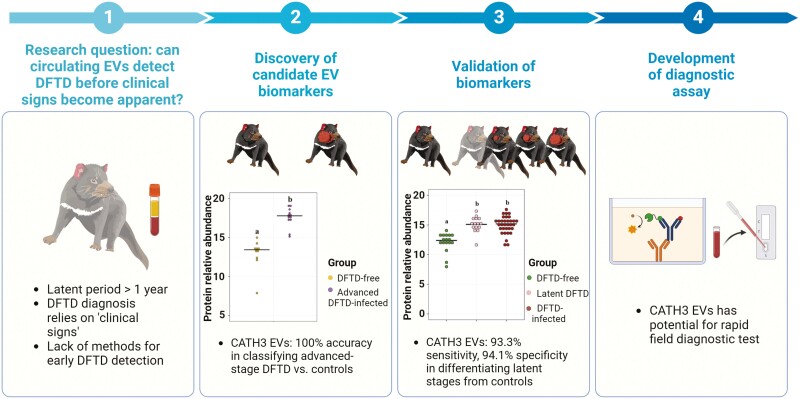
extracellular vesicle-based DFTD biomarker discovery process. Proteomic analysis of serum-derived EVs from Tasmanian devils identified the protein CATH3 as a remarkably accurate biomarker for distinguishing advanced-stage DFTD. Initial discovery work identified the protein CATH3 as a highly sensitive biomarker candidate. Subsequent validation further illustrated the potential of CATH3 in EVs to distinguish latent stages of infection from a healthy state. These findings can be used to develop an EV-based approach for rapid DFTD diagnostic testing in the field.

Research on Devil Facial Tumor Disease (DFTD), a transmissible cancer affecting Tasmanian devils, demonstrates how EVs can improve disease detection in the wild. DFTD is present in >90% of the Tasmanian devil’s range, resulting in an estimated 82% decline in wild devil populations since its discovery in 1996 [63]. Despite a latent period that can exceed one year, DFTD diagnosis primarily relies on visual tumor identification and biopsies [64]. However, during latent stages of infection, when tumors are absent, biopsies are not feasible. Consequently, a liquid biopsy, such as a blood sample, becomes necessary. Recently, analysis of EVs from the blood of DFTD-free and advanced DFTD-infected Tasmanian devils identified the protein cathelicidin-3 (CATH3) as a promising biomarker of DFTD, with 100% sensitivity and specificity in differentiating between DFTD-advanced individuals and controls [28]. Validation on an independent cohort of animals at various stages of infection, including latency (i.e. 3–6 months before tumors appear), further showed 93.8% accuracy in differentiating between latently infected individuals and healthy controls (Fig. 3). This work suggests that EV-based diagnostics may be particularly valuable for wildlife diseases with long latency periods. In human and lab studies, EV-based diagnostic biomarkers have been identified for diseases such as tuberculosis, HIV, and prion diseases [62, 65–67], paving the way for parallel studies in wildlife for similar diseases with notable latency periods, including bovine tuberculosis, feline immunodeficiency virus, and chronic wasting disease.

### Pathogenesis

Identifying the mechanisms underlying disease development, progression, resolution, or persistence (i.e. pathogenesis) is crucial for understanding the causes of disease and the factors leading to variable disease outcomes across hosts. EVs have been instrumental in this area, especially for understanding the pathogenesis of human diseases like HIV, where research shows that dendritic cells use EVs to transmit HIV particles to CD4+ T cells, facilitating within-host viral spread [68, 69]. Similarly, for Foot-and-mouth disease (FMD), a highly contagious viral disease affecting domestic and wild cloven-hoofed animals, research shows that EVs released from virus-infected porcine kidney cells contain full-length FMD viral genomic RNA and partial viral proteins that are capable of infecting both naïve cells and infant mice [70]. Moreover, unlike direct FMDV infections, which are effectively neutralized by FMDV-specific antibodies, EV-mediated infections are not fully neutralized, suggesting an immune evasion mechanism [70]. Given that wildlife can serve as reservoirs for FMD virus [71], applying similar EV-based approaches to investigate cell-to-cell virus transmission in wild hosts could uncover new mechanisms of within-host viral spread and persistence.

EV-based studies of other prominent livestock pathogens provide further insights applicable to wild species. For example, in Peste des petits ruminants (PPR), a viral disease affecting small ruminants, EVs from PPRV-infected goat peripheral blood mononuclear cells enhance the expression of signaling lymphocyte activation molecule (SLAM) receptors in recipient cells, boosting PPRV replication and spread [72]. Interestingly, SLAM is one of two host cell receptors involved in the pathogenesis of canine distemper [73], a disease affecting carnivores caused by a virus (CDV), which is closely related to PPRV. CDV infects a wide range of wild carnivores, many of conservation concern (e.g. Ethiopian wolf, African wild dog, and Giant Panda) [74]. Thus, exploring EVs in the context of CDV could yield new information about viral pathogenesis, a phenomenon that is not well understood in wildlife, especially CDV interactions with host cell receptors like SLAM [74].

Another wildlife disease of conservation concern where EVs could elucidate pathogenesis mechanisms is chytrid fungus (*Batrachochytrium dendrobatidis, Bd*) in amphibians. This fungus, linked to the decline of over 500 amphibian species worldwide, produces an array of small molecules that individually or synergistically facilitate pathogen evasion of the host immune system [75]. EVs from other fungal species, like *Candida spp* and *Cryptococcus neoformans*, contain virulence factors and other immunosuppressive compounds [76], suggesting that studying *Bd* EVs may reveal how *Bd* suppresses amphibian immunity to promote its own persistence.

### Resistance and tolerance

Host defense mechanisms against pathogens are crucial determinants of infection outcomes. Resistance and tolerance represent two main strategies hosts use for pathogen defense. Resistance involves effectively limiting pathogen growth or replication, while tolerance entails minimizing pathogen-induced damage [77]. Mechanisms of pathogen resistance and tolerance in wild animals are poorly understood, with most of the research to date concentrating on mechanisms of resistance [77, 78].

Human studies offer several examples of the role EVs play in modulating host resistance and tolerance, with potential relevance to wildlife. For instance, EVs secreted by HIV-1 infected CD4^**+**^ T cells carry the antiviral enzyme APOBEC3, which inhibits HIV-1 replication and confers HIV-1 resistance to EV recipient cells [79]. Similarly, in Hepatitis B (HBV), EVs facilitate cell-to-cell transmission of APOBEC3, leading to IFN-alpha-induced antiviral activity in HBV-infected cells [80]. Interestingly, a recent comparative genomic analysis of 37 bat species found evidence of APOBEC3 gene family expansion across multiple bat lineages [81]. Given the antiviral role of APOBEC3 enzymes, the diversification of this gene family may contribute to the extraordinary ability of some bats to coexist with viruses. Studying EVs to better understand links between APOBEC3 and host–virus interactions in bats could shed new light on mechanisms of antiviral resistance and tolerance in this group.

Research on teleost fish further demonstrates the importance of EVs in pathogen defense. A study on mandarin fish (*Siniperca chuatsi*) showed that serum EVs inhibit the replication of infectious spleen and kidney necrosis virus (ISKNV), an iridovirus threatening the aquaculture industry [82]. This inhibition was facilitated by the presence, within EVs, of myxovirus resistance 1 (Mx1) protein, which is known to have antiviral properties against iridoviruses [82]. Similarly, the transport of proinflammatory cytokines by host EVs provides another example of EV involvement in pathogen defense. For example, dendritic cells from mice infected with the zoonotic bacterium, *Chlamydia psittaci* release EVs bound to the cytokine TNF-alpha [83]. These TNF-alpha bound EVs then stimulate production of IFN-gamma in natural killer cells, suppressing the growth of *C. psitacci* in infected epithelial cells [83]. Interestingly, the EVs from infected dendritic cells lack bacterial components, suggesting a process mediated by host factors alone [83]. More generally, across various taxa ranging from plants to mammals, host-derived EVs have been shown to carry natural antimicrobial agents that combat fungal and bacterial pathogens, influencing infection outcomes [84]. Overall, these findings suggest that EVs contribute to host resistance against a wide range of pathogens, highlighting the potential utility of using EVs to uncover new mechanisms of pathogen defense in wildlife.

### Co-infection

Co-infection, the co-occurrence of multiple pathogens within a single host, is the norm rather than the exception in most animals [85]. Although interactions between co-infecting pathogens can significantly influence infection outcomes for hosts, the diversity of mechanisms underlying pathogen–pathogen interactions are only beginning to be uncovered and rarely in wild animal populations [86, 87]. Recent work suggests that pathogen-derived EVs may play a prominent role in orchestrating within-host pathogen interactions. A notable example is the interaction between *Moraxella catarrhalis* and *Haemophilus influenzae*, bacteria often co-infecting the human respiratory tract. In *in vitro* studies, OMVs derived from *M. catarrhalis* carry virulence factors (UsPA1 and UsPA2) that bind to the third component of the complement system (C3), inhibiting the complement cascade. This process promotes the survival of both *M. catarrhalis* and *H. influenzae* by inhibiting complement-dependent killing [88], revealing a clear EV-mediated mechanism by which one pathogen can facilitate another.

Similar EV-mediated interactions have been described between *Mycobacterium tuberculosis*, the causative agent of TB, and HIV-1 in humans. In this case, EVs secreted by host macrophages infected with *M. tuberculosis* can reactivate HIV-1 from latency by inducing oxidative stress [89]. This finding suggests an intriguing EV-associated mechanism that might help explain the disruptive effect of *Mycobacterium bovis* infection on within host-pathogen communities. *M. bovis*, a close relative of *M. tuberculosis*, is the causative agent of bovine tuberculosis (BTB), a globally distributed wildlife disease. Various species act as maintenance hosts for BTB in different geographic regions, from badgers and wild boar in Europe to white-tailed deer in North America and brushtail possums in New Zealand [90]. In African buffalo, the primary reservoir hosts for BTB in southern Africa, individuals who acquire *M. bovis* undergo a significant increase in the number and functional types of other pathogens they host [91]. Studying pathogen derived EVs may offer a new approach to begin unraveling how *M. bovis* exerts such broad effects on co-infecting pathogens. For example, screening EVs for pathogen-specific molecules released by host cells might provide insight into the presence and location of co-infecting pathogens within the host. Indeed, studies show that EVs released from *Mycobacterium bovis* bacillus Calmette-Guerin (BCG) and BCG-infected macrophages carry antigens like LAM and 19-kDa lipoprotein (LpqH) [92], that provide a signature of infection. Likewise, FMDV-infected cells secrete EVs containing most viral proteins [70], offering a distinct signature of infection from another common pathogen of buffalo.

EVs have also been shown to influence interactions during co-infection by expanding the range of infected cell types and enabling immune evasion. For instance, co-infection with Influenza A Virus (IAV) and Respiratory Syncytial Virus (RSV) in humans leads to the formation of EVs containing surface proteins from both viruses. IAV exploits these EVs, specifically the RSV surface glycoproteins packaged within them, to evade anti-IAV antibodies and infect cells lacking IAV receptors [93]. This mechanism could be relevant for understanding infection dynamics in wild species with viral coinfections. For example, in Australian flying foxes, simultaneous shedding of up to nine bat paramyxoviruses has been documented. Notably, pulses of multi-species viral shedding were common and also sporadically coincided with peak spillover of Hendra virus, an often fatal zoonotic pathogen [94]. Investigating whether EVs mediate virus interactions within zoonotic reservoir hosts, like these flying foxes, could provide insight into immunological drivers of spillover.

## Advantages and disadvantages of EVs in wild immunology

Incorporating the study of EVs into wild immunology research offers a way to navigate some significant logistical hurdles commonly encountered in wildlife work. These hurdles include but are not limited to: (i) difficulties inherent in capturing and immobilizing free-ranging animals for invasive sample collection; (ii) challenges associated with working in remote field locations lacking cold storage facilities or other major equipment; and (iii) the frequent lack of appropriate immunological reagents for assaying samples collected from non-model species.

The first major advantage of EVs is their presence across all body fluids and tissues [18]. For wildlife studies, where access to invasive samples such as blood and tissue can be limited, this feature provides invaluable flexibility. For example, EVs can be isolated from fluids such as feces and urine, providing a non-invasive option that increases the feasibility of conducting wildlife related immunological studies. Importantly, EVs isolated from different body fluids will carry distinct host- or pathogen-associated signals depending on the specific cell types present in those fluids [95]. The presence of pathogen-derived EVs in host body fluids will further depend on the colonization site of the pathogen and the trafficking pathway of EVs within the host [96]. Given the heterogeneous nature of the signals contained in EVs isolated from different sources, selecting the appropriate body fluid for EV isolation to match the research question of interest is critical. For example, in TB infected individuals, urinary EVs have been shown to contain mycobacterial-associated components [62], making urine an effective choice for detecting bacterial presence. In contrast, serum EVs tend to carry more host cell-derived signals, valuable for understanding the host’s immune response to TB infection [97].

The second major advantage of EVs is their stability under diverse conditions. Due in part to their bilipid membrane, EVs are resilient to high temperatures and harsh environments [98, 99]. This stability allows for effective storage under a variety of field conditions. For example, EVs in plasma can remain stable at temperatures ranging from 37°C to -20°C for up to three months, circumventing the need for short-term ultracold storage in remote settings [100]. For extended periods, a recent study recommended storage in PBS supplemented with human serum albumin and trehalose at −80°C to effectively preserve EV particle concentration, size, surface markers, RNA content, and functional integrity [101]. This capacity for long-term storage of EVs, while maintaining sample quality, is crucial for wildlife studies given often-limited access to sample processing equipment in remote field settings.

Once wildlife samples have been successfully collected and preserved, another persistent barrier to progress in wild immunology research has been the ongoing lack of immunological reagents for non-model species. Over the past decade, various ‘omics’ techniques have begun to help bridge this gap [102, 103]. However, applying omics methods to raw body fluids is often hampered by the presence of highly abundant molecules that can obscure the presence of low-abundance proteins. Therefore, a third major advantage of EVs stems from their ability to reduce this complexity and enrich a greater range of low-abundance proteins [98]. For instance, 99% of the protein content of serum/plasma is accounted for by just 22 highly abundant proteins, such as albumin and immunoglobulins [104]. In contrast, in EVs isolated from serum the presence of these abundant proteins can be completely reduced [105]. Additionally, by concentrating certain molecules, EVs can reveal biological pathways that are otherwise obscured in the original source material. A notable example is the detection of epithelial–mesenchymal transition (EMT) hallmark proteins in EVs derived from DFT2 cells, a second transmissible cancer affecting Tasmanian devils. The EMT pathway is thought to be involved in the pathogenesis of DFT2 and interestingly these proteins were not found in the proteome signatures of DFT2 cells themselves [106], indicating the capacity of EVs to concentrate molecules related to key disease processes.

Like all methodological tools, using EVs also presents challenges. Primary among these are issues related to standardizing methods for EV isolation [107]. Isolation techniques currently available for EVs vary significantly in terms of purity, yield, and practicality. Therefore, careful consideration must be given to the relative benefits and costs of each method, particularly in the context of the specific research questions of interest and work conditions. These considerations were thoroughly reviewed in a recent primer [108], providing guidance for making informed choices about EV isolation methods. For example, in the context of biomarker discovery, a methodology that ensures good yield, but which may not provide the purest samples (e.g. size exclusion chromatography techniques) might be preferred. Conversely, for understanding EV communication mechanisms, a method that prioritizes sample purity would be more suitable (e.g. density gradient) because it minimizes the co-isolation of molecules like lipoproteins, thus ensuring a more accurate attribution of observed mechanisms to EVs. Another challenge of using EVs is the lack of fully annotated genomes for many non-model species, which can hinder efforts to characterize EV molecular cargo. However, using databases from related species or computational methods like *de novo* assembly can help overcome this challenge [109].

**Figure 4:**
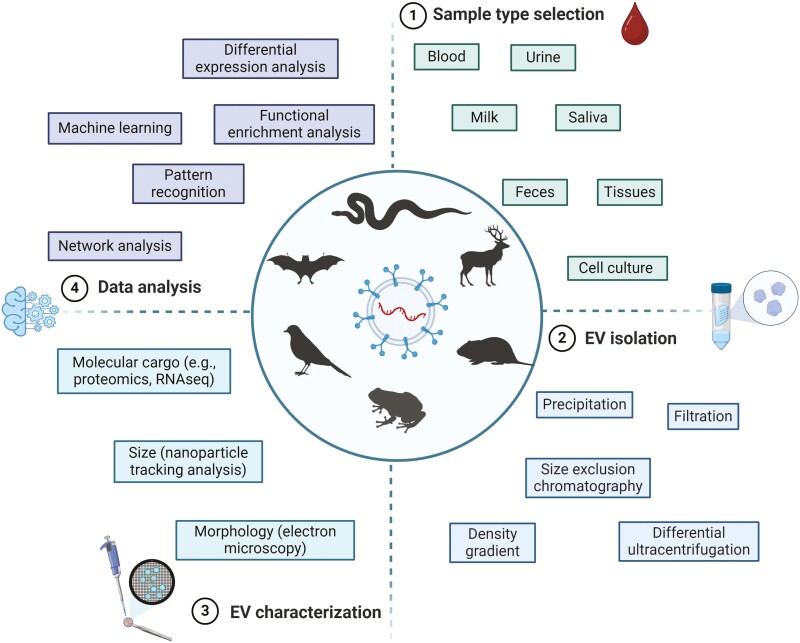
four essential steps for using extracellular vesicles (EVs) in wild immunology**. (**1) The first step involves selecting a sample type to be used as the EV source. This decision should be informed by the research question and the type of cellular communication being investigated. (2) The second step involves isolating EVs. The choice of method should depend on the sample type and work conditions. (3) The third step is the characterization of EVs, which includes quantifying their physical (size and morphology) and biochemical (protein, RNA, lipids) characteristics, with the specific approach tailored to the research question. (4) The fourth and final step is data analysis.

Overall, the advantages of using EVs in wild immunology research appear to outweigh the disadvantages. Moreover, the entry barrier to EV research is relatively low, making it accessible to a wide range of study types. For newcomers to EV research, resources like EV-TRACK [110] and the minimal information for studies of extracellular vesicles 2023 (MISEV2023) guidelines [111], are invaluable for providing methodological frameworks for standardization and repeatability. More generally, using EVs to address questions in wild immunology involves four essential steps (Fig. 4), each requiring careful decision-making that balances research goals with practical constraints. First, selecting the appropriate sample type for EV sourcing should align with the research question and the type of cellular communication under investigation. Blood samples are often used for studying systemic responses or diseases affecting the entire organism [112]. In contrast, saliva samples are particularly valuable for understanding local immune responses in the oral cavity [113]. Second, the choice of EV isolation method should consider sample type and work conditions. For example, size exclusion chromatography might be preferred for samples with high protein content, such as blood and urine, as it minimizes non-vesicular components like lipoproteins and offers medium practicality for field use, given access to electricity and a centrifuge [114]. The third step is lab-based characterization, including quantifying the physical (size and morphology) and biochemical (proteins, nucleic acids, lipids) characteristics of EVs. For biochemical characterization, different ‘omics’ techniques should be used based on the specific molecular cargo of interest. For proteins, proteomics approaches such as mass spectrometry are used to comprehensively profile protein contents [14]. For nucleic acids, genomics and transcriptomics analyses can be performed using next-generation sequencing, which allows for a broad profiling of DNA and RNA sequences within EVs [14]. The fourth and final step is data analysis, where specific methods (e.g. pattern recognition, differential expression analysis, machine learning, and various statistical and bioinformatic approaches) should be tailored to the attributes of the dataset and study goals [115, 116].

## Conclusion

Wild immunology exploits the vast genetic and environmental variability of wild species to provide insights into fundamental and translational immunological questions. Despite its immense potential, the field faces logistical constraints, including challenges with sample collection and storage in field settings, as well as a persistent lack of essential reagents for immune marker detection and quantification. EV biology holds untapped potential to help address these challenges and enhance our understanding of immunology in wild populations. The universal presence of EVs across species and cell types renders them highly suitable for studying diverse non-model organisms. Furthermore, their resilience under extreme conditions positions EVs as a formidable tool for navigating key logistical hurdles of wildlife research. Most significantly, EVs reflect the biological state of their originating cells and encapsulate and transport biomolecules for intricate intercellular communication, a function critical for decoding complex immune system interactions. Leveraging these attributes of EV biology in wild immunology can have a profound impact on the field.

## Data Availability

Not applicable.

## Abbreviations

BCG: bacillus Calmette-Guerin
*Bd*: *Batrachochytrium dendrobatidis*
BTB: bovine tuberculosis
CATH3: cathelicidin-3
CDV: canine distemper virus
DFTD: Devil Facial Tumor Disease
EMT: epithelial–mesenchymal transition
EVs: Extracellular vesicles
FASL: Fas ligand
FMD: Foot-and-mouth disease
HB: Hepatitis B
IAV: Influenza A Virus
iRBCs: infected red blood cells
ISKNV: infectious spleen and kidney necrosis virus
LAM: lipoarabinomannan
LpqH: 19-kDa lipoprotein
LUCA: Last Universal Common Ancestor
MAMPs: microbe-associated molecular patterns
MHC: major histocompatibility complex
MISEV2023: minimal information for studies of extracellular vesicles 2023
MV: membrane vesicles
Mx1: Myxovirus resistant 1 protein
OMVs: outer membrane vesicles
PDL1: Programmed Cell Death ligand1
PPR: Peste des petits ruminants
RSV: Respiratory Syncytial Virus
SLAM: signaling lymphocyte activation molecule
STING: Stimulator of Interferon Genes
TB: tuberculosis

## Permission to reproduce

Graphs included in Figure 3 originate from the following publication: https://doi.org/10.3389/fimmu.2022.858423. These graphs are used under the Creative Commons CC-BY license (version 4.0), which allows for free use, distribution, and adaptation with appropriate attribution.
